# Epstein-Barr Virus-Related Hemophagocytic Lymphohistiocytosis: Hematologic Emergency in the Critical Care Setting

**DOI:** 10.1155/2015/491567

**Published:** 2015-02-10

**Authors:** Neda Hashemi-Sadraei, Pimprapa Vejpongsa, Muhamed Baljevic, Lei Chen, Modupe Idowu

**Affiliations:** ^1^Department of Internal Medicine, University of Texas Health Science Center at Houston, Houston, TX 77030, USA; ^2^Division of Cancer Medicine, The University of Texas, MD Anderson Cancer Center, Houston, TX 77030, USA; ^3^Department of Pathology and Laboratory Medicine, University of Texas Health Science Center at Houston, Houston, TX 77030, USA

## Abstract

Hemophagocytic lymphohistiocytosis (HLH) is a rare and potential life-threatening clinical syndrome that results from uncontrolled activation of the immune system. Secondary HLH, more commonly observed in adult patients, is seen in the context of underlying triggering conditions. Epstein-Barr virus (EBV) has been recognized as the leading infectious cause and is associated with a poor outcome. As clinical and laboratory features of HLH could overlap with septic shock syndrome in most patients, the diagnosis of HLH, especially in adults, is the most challenging aspect of the disease that results in delayed recognition and treatment of rapidly progressive multiorgan system failure. We report a case of Hemophagocytic lymphohistiocytosis in a patient who presented with signs of septic shock syndrome and we review the literature on the topic.

## 1. Introduction

Hemophagocytic lymphohistiocytosis (HLH) is a rare life-threatening syndrome of immune dysregulation typified by extreme immunologic response and tissue infiltration of cytokine-activated tissue macrophages (histiocytes) as well as cytotoxic T-lymphocytes that can lead to multiorgan system failure. It can sometimes occur as a primary disorder traditionally described in pediatric population where it is usually driven by autosomal recessive gene defects of granulocyte-associated cytotoxic pathways, ultimately resulting in exuberant T-cell and macrophage response that is deregulated, causing indiscriminate phagocytosis of peripheral blood elements of resident tissues [1, 2].

Secondary HLH, more commonly observed in adult patients, is seen in the context of underlying triggering conditions ranging from inflammatory processes such as autoimmune and rheumatologic disorders and neoplastic disorders particularly hematologic malignancies such as lymphoid cancers and leukemias to various forms of commonly observed, but not limited to, viral infections. Triggering infections are usually viral in etiology, especially the herpesviridae family, human immunodeficiency virus (HIV), adenovirus, and parvovirus. Less commonly, bacteria, fungi, and parasites have been reported as well. Epstein-Barr virus (EBV) has been recognized as the leading infectious cause [3, 4].

The diagnosis of HLH is based on fulfilling the diagnostic criteria described in the HLH-2004 protocol [5]; however, not all patients with HLH fulfill the criteria. In order to avoid delayed diagnosis which often leads to delays in initiation of therapy, hence higher mortality, modified diagnostic criteria have been proposed [6]. Although hemophagocytosis represents an integral part of the diagnostic criteria, it is not required to make the diagnosis of HLH based on either of the two criteria.

A standard established treatment for HLH is described in the HLH-94 protocol and later modified in HLH-2004. Principles of treatment include elimination of the trigger and suppression of the inflammation and activated immune cells, as well as supportive measures [5, 7]. Nonetheless, treatment of HLH is challenging and mortality from HLH remains high. Patients are at increased risk of infections and should be monitored very closely during treatment and a subsequent follow-up [8].

## 2. Case Report

A 40-year-old Hispanic man with no significant medical history presented with sharp right upper quadrant and epigastric pain, dizziness, and fever as high as 102 Fahrenheit for 4 days. He also reported vomiting over 10 times a day and severe green watery diarrhea multiple times a day. Three weeks earlier he was seen by a primary care physician for cellulitis of the lower extremities and was prescribed empiric antibiotic course. Given worsening symptoms, he presented for admission, and he was found to be febrile (102 F), hypotensive (79/54 mmHg), and tachycardic (132 bpm). On physical examination he had icteric skin, tenderness on right upper quadrant, and right lower extremity swelling with mid-tibial erythema. The rest of the physical exam was unremarkable.

Laboratory workup showed white blood cell (WBC) 2500/*μ*L, hemoglobin (Hgb) 10.9 g/dL, platelet count 25,000/*μ*L, prothrombin time 14.9 seconds; partial thromboplastin time 60 seconds, d-dimer 6.19 ug/mL, and fibrinogen 180 mg/dL, serum creatinine 2.9 mg/dL, blood urea nitrogen (BUN) 75 mg/dL, alanine aminotransferase (ALT) 373 U/L, aspartate aminotransferase (AST) 669 U/L, alkaline phosphatase 119 U/L, total bilirubin 4.3 mg/dL (direct bilirubin 3.0 mg/dL), normal lipase, triglyceride 378 mg/dL, and lactate dehydrogenase (LDH) 2,829 U/L (Table 1).

On abdominal ultrasound, his liver measured 16.3 cm with no focal lesions; spleen was mildly enlarged at 13.7 cm. He was admitted directly to the intensive care unit for presumed septic shock and early goal-directed therapy. Intravascular fluid was started, blood cultures were sent, and broad-spectrum antibiotics (vancomycin, cefepime, azithromycin, and metronidazole) were initiated. Abdominal/pelvic computed tomography (CT) without contrast revealed an enlarged spleen and no further abnormalities.

**Figure 1:**
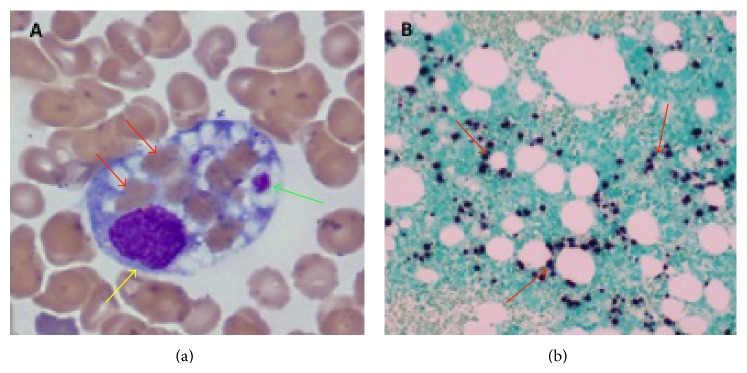
Images of the bone marrow. (a) Bone marrow aspirate smear, 1000x magnification, shows a hemophagocyte which engulfed 7 separate red blood cells (red arrows) and 2 platelets (green arrow). Yellow arrow points to a nuclei of histiocyte; (b) bone marrow clot section, EBV-encoded RNA (EBER) staining of the bone marrow revealing extensive EBV infected lymphocytes in the background (dark nuclear stain).

**Figure 2:**
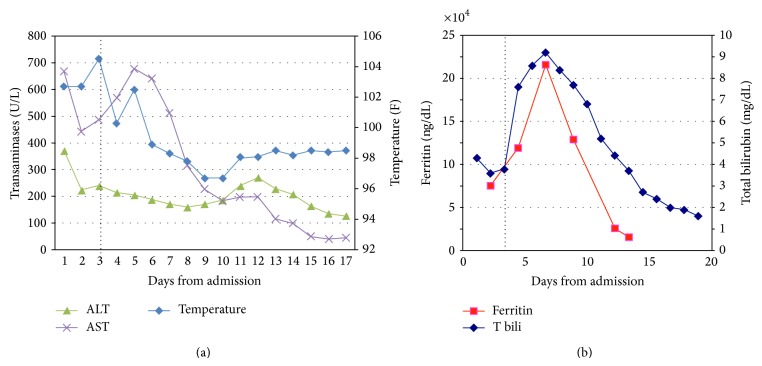
Trend of liver enzymes, temperature (a), total bilirubin, and ferritin (b) during the initial hospital stay. Dotted line indicates the start of therapy on day 3 (ALT: alanine aminotransferase; AST: aspartate aminotransferase; T bili: total bilirubin).

Hematology service was consulted for evaluation of pancytopenia on day 2 given the decrease in WBC and Hgb to 1,400/*μ*L and 8.9 g/dL, respectively. Peripheral blood smear showed numerous echinocytes, few ovalocytes, and no schistocytes. Routine serum iron was 139 ug/dL with iron saturation 55%, while serum ferritin measured 72,285 ng/dL. Given that initial blood and urine cultures were negative at that point, our clinical suspicion for HLH was very high. After careful assessment of the diagnostic criteria, 5 out of 8 were met by our patient (fever, pancytopenia, high triglycerides, elevated ferritin, and splenomegaly). Bone marrow biopsy was performed on day 3 and it revealed 3 hemophagocytes (Figure 1(a)), further confirming the diagnosis. Therapy for HLH was started on the same day by the model of HLH-94 protocol with dexamethasone 20 mg IV daily and 75% dose-reduced etoposide twice a week due to liver and kidney dysfunction. Doxycycline 100 mg PO every 12 h was added for coverage of atypical microorganisms. Patient's daily liver transaminases and temperature are represented on Figure 2(a) and in Table 1. Variation of the serum ferritin level and total bilirubin during the hospitalization is shown on Figure 2(b) and in Table 1.

A comprehensive rheumatologic and infectious workup was sent to identify any potential underlying triggers of HLH. Rheumatologic workup included anti-Sm, anti-Scl-70, ANA, ANCA, anti-dsDNA, anti- RNP, anti-cardiolipin, anti-SSA, and anti-SSB antibodies, all of which were negative. Infectious workup was negative for cytomegalovirus, herpes simplex viruses 1 and 2,* Brucella*, parvovirus B19, cryptococcus,* Histoplasma*, HIV, and hepatitis B and hepatitis C. On day 5, Serum EBV IgM was found positive and the patient was started on acyclovir 5 mg/kg IV every 24 h. Cefepime, vancomycin, and metronidazole were discontinued. On day 8, serum EBV PCR (qualitative test) was reported positive also. Review and EBER staining of the bone marrow revealed extensive infiltration of the marrow by the EBV (Figure 1(b)). Following the administration of HLH-94 therapy as well as IV Acyclovir, the patient's condition started improving and his fever subsided. Rituximab was not given for EBV infection due to the dramatic nature of his improvement on the initial therapy (dexamethasone, Etoposide, and antimicrobials).

On day 9, Q fever phase II serology was found to be positive at 1 : 64 titers. On further questioning, patient recalled having had a contact with his dog that had delivered puppies 3 months prior to development of his symptoms. Ciprofloxacin 500 mg PO daily was added. Patient continued with progressive clinical improvement and, on day 11, he was transferred out of the ICU to the medical floor for further management.

He was found to have acute tubular necrosis from systemic involvement of HLH and required intermittent hemodialysis for metabolic optimization. By day 17, patient had completed 14 days of IV acyclovir, 4 doses of etoposide, 14 days of doxycycline, and 9 days of ciprofloxacin. Repeat Q fever phase II serology was negative. On day 17, patient was discharged home on ciprofloxacin, further acyclovir course, fluconazole, trimetoprim/sulfamethoxazole, and steroid taper.

He continued to show improvement on outpatient laboratory follow-up visits. On hematology follow-up visit on day 28, renal function and liver function showed improvement and his blood cell counts showed full recovery (Table 2). On the same visit, acyclovir, fluconazole, and ciprofloxacin were discontinued given resolution of neutropenia. Prophylactic trimetoprim/sulfamethoxazole was continued along with dexamethasone.

On day 35, patient presented with shortness of breath and acute lower extremity pain. His vital signs were stable. He had scleral icterus and his strength was decreased in all extremities. Laboratory investigations showed thrombocytopenia (48,000/*μ*L), high ferritin (4,963 ng/dL), and elevated transaminases (Table 2). CT angiogram of the chest did not show pulmonary embolism. Patient was admitted and supportive measures with oxygen, electrolyte replacement, and pain management were initiated. By the next day his respiratory distress worsened, he became hypotensive, liver function deteriorated progressively, and platelet count dropped to 24,000/*μ*L (Table 2). He developed severe metabolic acidosis with serum pH 7.2, bicarbonate 13 mmol/L, and lactic acid 11 mmol/L. Blood cultures were drawn and patient was started on a broad-spectrum empiric antibiotic coverage with vancomycin, cefepime, and metronidazole. He was transferred to medical ICU and was intubated for airway protection. Gancyclovir was added for potential EBV-associated hepatitis given prior documentation of EBV-related HLH. Despite these measures, he progressed to fulminant hepatic failure with significant distributive shock. Given the patient's high ferritin, though significantly better than previous ferritin of 15,550 ng/dL on day 12, thrombocytopenia, and negative urine and blood cultures, he was treated with high dose methylprednisolone (1 g/d) and intravenous immunoglobulin (1 g/kg) for possible relapsed or refractory HLH, while etoposide was omitted due to fulminant liver failure (bilirubin 10.5 mg/dL).

On the evening of day 2 of readmission, he developed cardiac arrest due to ventricular tachycardia and cardiopulmonary resuscitation was unsuccessful. His autopsy revealed multiorgan failure consistent with clinical history of severe lactic acidosis, shock, and occasional cells suspicious of hemophagocytosis in the bone marrow.

## 3. Discussion

HLH is a rare and potential life-threatening clinical syndrome that results from uncontrolled activation of the immune system. Historically, HLH is more common in children or adolescences; however, adult onset of HLH is becoming increasingly more recognized [9]. Currently, the precise pathophysiology of HLH remains unclear. Dysregulation of cytotoxic T cells and natural killer cells that lead to activation and proliferation of histiocytes with excessive cytokine release has been proposed as the underlying pathophysiologic mechanisms [10]. Primary HLH is a familial disorder that commonly occurs in children with predisposed genetic defects involving lymphocytic granule-mediated cytotoxic pathway [11]. Secondary HLH is an acquired syndrome that most commonly occurs in adulthood and is usually triggered by an underlying malignancy, autoimmune disease, or an infection [3]. Etiologies associated with secondary HLH are described in Table 3.

Recent studies suggest that late-onset familial HLH occurs more commonly than was suspected previously. In a retrospective review of 175 adults (age range 18 to 75 years) with clinical diagnosis of HLH, 14% were found to have gene mutations, most commonly defects in Perforin gene [12]. EBV, the most common infectious cause of secondary HLH, may also trigger HLH in patients with any form of familial disease. Despite this, HLH in adults is commonly attributed to an infectious etiology and a full genetic workup may not be performed [13]. Given the adult age of our patient, lack of corroborating or suspicious family history for HLH, and extensive infiltration of the bone marrow by EBV as the likely culprit as well as conscious choice to adopt a judicious use of health care resources, genetic testing was not undertaken for our patient.

As clinical and laboratory features of HLH could overlap with septic shock syndrome in most patients, the diagnosis of HLH, especially in adults, is the most challenging aspect of the disease that results in delayed recognition and treatment of rapidly progressive multiorgan system dysfunction/failure [14]. Early recognition and prompt treatment is critical and could be a key to prevention of fatal outcomes in HLH. A retrospective study in the critical care setting suggested that the HLH 2004 criteria might not be a reliable tool to differentiate between HLH and sepsis syndrome [15]. Some case reports suggest that ferritin level could be a useful test in this clinical scenario; when higher than 10,000 ng/mL, it is 90% sensitive and 96% specific for HLH [16]. Ferritin, an acute phase reactant, is a nonspecific biomarker that could be elevated in many circumstances. However, the ferritin level higher than 10,000 ng/mL is known to be associated with only a few other disorders such as systemic juvenile rheumatoid arthritis, adult still's disease, and histiocytic malignancies [17, 18]. In the appropriate clinical setting, extremely high ferritin level should alert physicians to search for other clinical and laboratory parameters to determine if the diagnostic criteria for HLH are met. The concentration of circulating soluble interleukin- (IL-) 2 receptor, also known as soluble CD25, is a very helpful diagnostic tool in HLH [19]. Since soluble IL-2 receptor reflects degree of T-cell activation, it can also be used to evaluate severity of the disease during follow-up period. Unfortunately, at present, this test is not readily available, is very expensive, and usually takes up to 2 weeks to process [20].

Two HLH treatment protocols, HLH-94 and HLH-2004, were developed based on the experience in children with primary HLH [5, 7]. These treatments include dexamethasone, etoposide, cyclosporine, and intravenous immunoglobulin. Although it remains unclear whether these protocols are the appropriate treatments for adults with secondary EBV-related HLH, most physicians refer back to them for initial therapeutic choice. Some studies suggested that early initiation of etoposide provided survival benefit in adults with secondary HLH [21, 22]. Risk and benefit of etoposide in this group of patients should be carefully evaluated given that severe liver and renal dysfunctions are commonly encountered in HLH patients. In patients with HLH and multiorgan failure, successful treatments with reduced-dose etoposide with close monitor have been reported [23]. In contrast, a few reports advocate more conservative and supportive management with corticosteroid/cyclosporine and suggested that it might be adequate in some patients [24, 25]. In our patient, dose-reduced etoposide was given along with steroid therapy during the initial hospitalization, which led to a striking improvement in the liver function and clinical status of the patient. On the second admission, due to his rapid and fulminant liver failure, etoposide was omitted and patient was given high dose steroids with IVIG, however unfortunately without benefit. Given the improvement in ferritin compared to his previous admission, he likely had septic shock, which might have resulted in exacerbation or relapse of his HLH. Patient's clinical and laboratory data together with autopsy report were also consistent with septic shock with multiorgan failure.

In a 16-year literature review of HLH cases in children up to 18 years of age, of the 198 cases diagnosed with infection-associated HLH, 52% died, mostly from severe infections, organ failure, or disseminated intravascular coagulation. EBV was found to be associated with the worst outcome (73% mortality) [3]. In retrospective studies, high EBV-DNA viral load (>1,000 copies/mL), hyperbilirubinemia (>1.8 mg/dL), and highly elevated ferritin level (>20,300 ng/mL) were proposed as factors associated with poor outcome in patients with EBV-related HLH [26, 27]. Our patient had at least two of the above factors (bilirubin and ferritin levels) making his prognosis unfavorable. Rituximab can be effective for EBV infection [28], but, due to our patient's rapid improvement with the initial therapy, we did not feel the need to treat him with this agent at that time. However, if he did not improve with our initial regimen or if he had evidence of worsening EBV infection, we would have offered this therapy to him.

Although mortality rate of HLH has been reported as high as 52%, rapid initiation of treatment can possibly reverse the devastating course of the disease [3]. Given the extremely high ferritin level found in our patient, diagnosis was made relatively promptly and appropriate therapy was initiated on the 3rd day of hospitalization. We believe checking ferritin level in all patients with clinical picture of sepsis could help raise initial suspicion index, which would trigger urgent involvement of hematology subspecialty team that can attempt to make a timely diagnosis. We therefore suggest that a screening ferritin level be included in early sepsis protocols; if ferritin level is found to be above 10,000, hematology consultation should be sought urgently in order to identify the cases of HLH in a timely manner.

Unfortunately the reported patient returned with severe sepsis and possible relapse of HLH, succumbing to the disease in short period of time. We therefore believe that patients should be followed up very frequently and with earnest vigilance with continuation of broad-spectrum prophylactic antibiotics even in the absence of neutropenia until the full spectrum of organ function is restored.

## 4. Conclusion

Despite being primarily a pediatric disorder, HLH does also occur in adults, particularly secondary to an underlying triggering condition. HLH is a true hematologic emergency; prompt diagnosis and treatment are crucial to avoid imminent multiorgan dysfunction resulting in a fatal outcome. Checking ferritin level as part of a standard sepsis protocols can help in raising the initial index of suspicion as it did in our case, guiding the medical care providers to seek an emergent hematology consultation for early initiation of treatment.

## Figures and Tables

**Table 1:** Trend of liver function test, ferritin levels, blood counts, and creatinine during the initial hospital stay.

Days from admission	1	2	4	6	8	12	17
Temperature (F)	102.7	102.7	100.3	98.9	97.8	98.1	98.5
ALT (U/L)	373	225	214	188	160	270	126
AST (U/L)	669	444	568	640	314	198	45
ALP (U/L)	119	59	79	169	236	340	318
Total bilirubin (mg/dL)	4.3	3.6	7.6	9.2	7.7	3.7	1.6
Ferritin (ng/dL)	—	75285	119440	216048	128888	15550	—
Creatinine (mg/dL)	2.9	2.4	2.3	5.6	5.1	3.9	4.5
WBC (/*μ*L)	2500	1400	2200	1500	900	1400	900
ANC (/*μ*L)	2240	1300	2090	1370	830	1210	730
Hgb (g/dL)	10.6	8.9	9.4	7.3	6.7	6.8	7.9
Platelet (/*μ*L)	25000	20000	18000	30000	33000	47000	77000

ALT: alanine aminotransferase; ALP: alkaline phosphatase; ANC: absolute neutrophil count; AST: aspartate aminotransferase; Hgb: hemoglobin; WBC: white blood cells.

**Table 2:** Trend of laboratory work after discharge and during the second hospital stay.

Days from initial admission	18	21	28	35	36
				Second admission	
ALT (U/L)	128	110	108	383	3657
AST (U/L)	44	35	38	296	6197
ALP (U/L)	287	279	232	341	418
Total bilirubin (mg/dL)	1.7	1.5	1.1	2.3	10.5
Ferritin (ng/dL)				4963	
Creatinine (mg/dL)	4.5	3.6	1.7	0.8	1.2
WBC (/*μ*L)	900	1100	3800	7500	10000
ANC (/*μ*L)	650	690	2550	62600	7900
Hgb (g/dL)	8.3	7.8	8.1	8.0	9.1
Platelet (/*μ*L)	113000	158000	197000	46000	24000

ALT: alanine aminotransferase; ALP: alkaline phosphatase; ANC: absolute neutrophil count; AST: aspartate aminotransferase; Hgb: hemoglobin; WBC: white blood cells.

**Table 3:** Etiologies associated with secondary HLH.

Malignancy	
Hematologic	Lymphoma, multiple myeloma, ALL
Nonhematologic	Prostate cancer, lung cancer, hepatocellular carcinoma
Autoimmune disease	Systemic juvenile, SLE, Kawasaki disease, seronegative spondyloarthropathies
Infection	
Bacterial	*Coxiella burnetii, Staphylococcus aureus*, *Campylobacter* spp., *Chlamydia* spp., *Legionella* spp., *Mycobacterium* spp., *Mycoplasma* spp., *Salmonella* spp., *Brucella* spp., *Ehrlichia* spp., *Borrelia* spp., *Clostridium* spp., *Listeria* spp.
Fungal	*Candida* spp., *Cryptococcus* spp., *Histoplasma* spp., *Pneumocystis* spp., *Aspergillus* spp., *Fusarium* spp.
Parasitic	*Plasmodium* spp., *Toxoplasm*a spp., *Babesia* spp., *Strongyloides* spp., *Leishmania* spp.
Viral	Herpes virus (EBV, CMV, HHV-8, HSV), HIV, HTLV, HAV, HBV, HCV, HEV, Influenza, mumps, measles, rubella, dengue, hantavirus, parvovirus B19, enterovirus

ALL: acute lymphocytic leukemia; SLE: systemic lupus erythematosus; EBV: Epstein-Barr virus; CMV: cytomegalovirus; HHV-8: human herpesvirus 8; HSV: herpes simplex virus; HIV: human immunodeficiency virus; HTLV: human T-lymphotropic virus; HAV: hepatitis A virus; HBV: hepatitis B virus; HCV: hepatitis C virus; HEV: hepatitis E virus.
